# Efficacy of concomitant acromioplasty in the treatment of rotator cuff tears: A systematic review and meta-analysis

**DOI:** 10.1371/journal.pone.0207306

**Published:** 2018-11-15

**Authors:** Cong Cheng, Bin Chen, Hongwei Xu, Zhongwei Zhang, Weibin Xu

**Affiliations:** Orthopedics Department, The Second Affiliated Hospital of Jiaxing University, Jiaxing, Zhejiang Province, China; Sint Antonius Ziekenhuis, NETHERLANDS

## Abstract

**Background:**

Scientific evidence is not clear regarding the routine use of acromioplasty in the treatment of rotator cuff repair. The aim of this study was to compare clinical outcomes between patients undergoing arthroscopic rotator cuff repair with and without concomitant acromioplasty.

**Methods:**

Medline, Cochrane Library, and EMBASE databases were searched to identify eligible studies focused on arthroscopic rotator cuff repair with and without acromioplasty from January 2000 to February 2018. Postoperative functional outcomes, visual analog scale (VAS) for pain and reoperation rate were extracted for systemic analysis.

**Results:**

Six randomized controlled trials (RCTs) and one cohort study (CS), including 651 patients, fulfilled our selection criteria. The results showed a significant difference in American Shoulder and Elbow Surgeons (ASES) score, but not in the Constant score, University of California-Los Angeles (UCLA) score, or Simple Shoulder Test (SST) score, in the treatment of rotator cuff tear with or without concomitant acromioplasty at the final follow-up. In the subgroup analysis, the results showed no significant differences between the two treatments in reoperation rate at the final follow-up or VAS score at 6 months postoperatively and final follow-up, but there was a significant difference in VAS score at 12 months postoperatively in favor of acromioplasty treatment. The evidence quality for each outcome evaluated by the GRADE system was low.

**Conclusions:**

In summary, our present study demonstrated that acromioplasty treatment is significantly superior to nonacromioplasty in shoulder pain relief at 12 months postoperatively and in ASES score improvement at the final follow-up in conjunction with rotator cuff repair. However, these significant differences were not clinically relevant. Thus, there were no differences in shoulder function or pain scores for patients undergoing rotator cuff repair with and without acromioplasty. Further high-quality studies with larger sample sizes and long-term follow-ups are needed to clarify this issue.

## Introduction

Armstrong et al.[1] first observed mechanical impingement between the rotator cuff and acromion in shoulder impingement syndrome in 1949. Subsequently, Neer et al.[2] reported that bony spurs at the anterior and lateral edges of the acromion led to 95% of rotator cuff attritions and tears, and they then performed acromioplasty as a concomitant procedure with or without rotator cuff tear, which has recently evolved into an arthroscopic approach[3]. The subsequent studies by Balke and Worland et al.[4,5] described a relationship between acromial morphology and the presence of rotator cuff pathologies, and they found that a hooked-type acromion was more likely to cause rotator cuff disease. Then, Ellman et al.[6] developed the arthroscopic subacromial decompression (ASD) treatment, which included acromioplasty, coracoacromial ligament (CAL) resection, and subacromial bursectomy, to heighten the subacromial space and protect the integrity of the rotator cuff. Since then, arthroscopic acromioplasty and arthroscopic subacromial decompression (ASD) have gained large popularity. From 1996 to 2008, the number of acromioplasty treatments increased by approximately 250%[7]. Nearly 40% of patients undergoing rotator cuff repairs in Finland received arthroscopic acromioplasty[8].

Arthroscopic acromioplasty is increasingly recognized by surgeons due to its concomitant visualization of the glenohumeral joint, preservation of the deltoid muscle, improvement of subacromial sight, and quick recovery time [9,10]. However, several studies have suggested that functional outcomes of rotator cuff repair are similar whether or not acromioplasty is used. Ranalletta et al.[11] reported significant functional improvements in seventy-four patients undergoing arthroscopic rotator cuff repairs at the midterm follow-up of 42 months without acromioplasty. Freedman et al.[12] found that the improvement of clinical outcomes and pathologic progress of rotator cuff were irrelevant to concomitant arthroscopic acromioplasty at the mean follow-up of 4.5 years. Thus, the benefits of acromioplasty remain questionable, although it has been routinely performed for many years [13]. Moreover, several studies have indicated that rotator cuff tears are mainly due to age-related degeneration and intrinsic overloading rather than extrinsic factors, such as subacromial impingement [14,15].

To our knowledge, previous systematic reviews have not supported the routine use of acromioplasty in conjunction with rotator cuff repair [16–19]; rather, they have recommended that further studies be performed. To date, several new studies have been published to assess the role of acromioplasty, which warrants an updated review containing all clinical trials.

The purpose of this study was to systematically evaluate the available functional scores and reoperation rate in clinical trials to ascertain the efficacy of acromioplasty in patients with rotator cuff repair. These results will provide more reliable evidence to determine the appropriate method.

## Materials and methods

### Literature search

This systematic review and meta-analysis was conducted in accordance with PRISMA guidelines (data in S1 Table). Medline, Cochrane Library, and EMBASE databases were searched by two independent investigators with no language restrictions from January 2000 to February 2018 (data in S1 Appendix). We used a text search strategy with combinations of the following terms: (rotator cuff) AND (acromioplasty OR subacromial decompression). Reference lists were also hand-searched for relevant studies.

### Inclusion and exclusion criteria

Two independent reviewers screened article titles and abstracts based on the following inclusion criteria: (1) randomized controlled trials (RCTs) and cohort studies comparing acromioplasty or subacromial decompression with nonacromioplasty; (2) studies of patients diagnosed with rotator cuff tears; (3) studies that provided quantifiable outcomes. The following exclusion criteria were used: (1) studies that did not meet the inclusion criteria; (2) animal studies, case reports, comments, conference papers, meta-analysis or systematic reviews.

### Data extraction

Three independent reviewers designed a structured table and collected all of the relevant data into a database. The following information was extracted from each study that met the inclusion criteria: first author’s name, quality evaluation, inclusion criteria, surgical procedures, sample size, mean follow-uptime, follow-up rate, and outcomes measures. We also attempted to contact the corresponding authors to verify the accuracy of the data and to obtain further analytical data.

### Quality assessment

The methodological quality of each RCT was assessed using the software RevMan (version 5.1, The Cochrane Collaboration, Oxford, England) by two reviewers, which contained the following items: random sequence generation, allocation concealment, blinding, incomplete outcome data, selective reporting, and other sources of bias. It was judged by answering a question, with “yes” indicating low risk of bias, “no” indicating high risk of bias, and “unclear” indicating unclear or unknown risk of bias [20]. The methodological quality of cohort studies were assessed via the Newcastle-Ottawa Scale (NOS) by the same reviewers. The corresponding author was consulted when there were any disagreements, and a consensus was reached by discussion.

### Evidence synthesis

The evidence grade for the main outcomes are assessed using the guidelines of the Recommendations Assessment, Development and Evaluation (GRADE) system working group including the following items: risk of bias, inconsistency, indirectness, imprecision and publication bias. The recommendation level of evidence is classified into the following categories: (1) high, which means that further research is unlikely to change confidence in the effect estimate; (2) moderate, which means that further research is likely to significantly change confidence in the effect estimate but may change the estimate; (3) low, which means that further research is likely to significantly change confidence in the effect estimate and to change the estimate; and (4) very low, which means that any effect estimate is uncertain. The evidence quality is graded using the GRADEpro Version 3.6 software. The evidence quality was graded using the GRADEpro Version 3.6 software. The strengths of the recommendations were based on the quality of the evidence.

### Statistical analysis

All analyses were performed by RevMan 5.1. For continuous data, standardized mean differences (SMD) or weighted mean (WMD) differences were calculated with 95% confidence intervals (CIs). For dichotomous data, relative risk (RR) and 95% CIs were used as a summary statistic. A p-value < 0.05 was considered statistically significant. The p-value with the Cochrane Q-test was texted, and the I^2^ statistic was used to measure the inconsistency of treatment effects across studies. A random effects model was used if high heterogeneity was detected (p<0.10, I^2^>50%); otherwise, a fixed effects model was used. Subgroup analyses were conducted to evaluate the stability of the results. Sensitivity analyses were performed by the leave-one-out approach using STATA 14.0 (STATA Corp, College Station, TX). Publication bias was evaluated by a funnel plot if more than 10 studies were included [21].

## Results

### Study identification and selection

**Fig 1:**
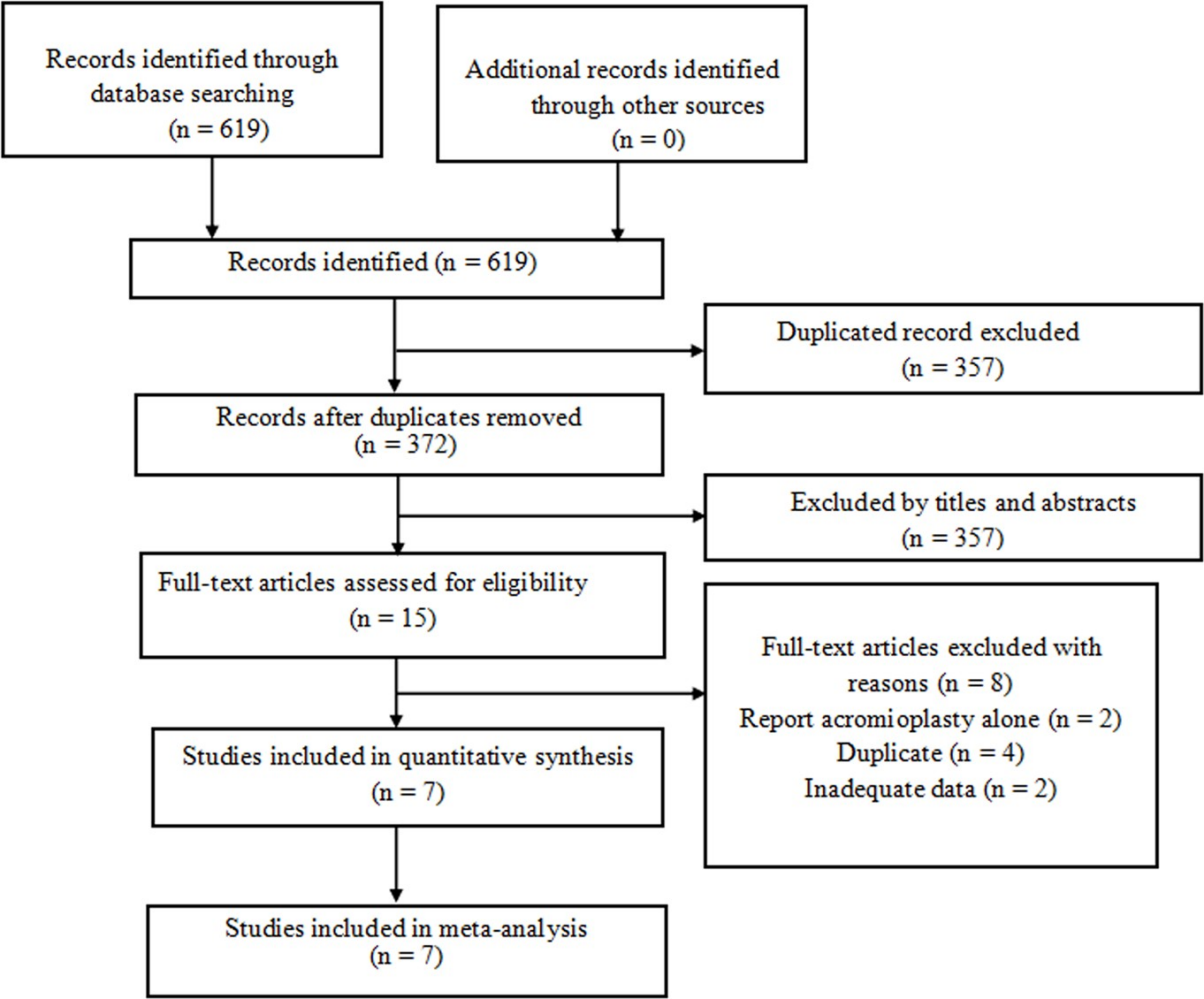
The flow diagram of study selection.

In total, 619 candidate publications were retrieved. However, 347 publications were excluded due to duplications. Among the 372 remaining articles, 357 articles were excluded according to titles and abstracts. Then 15 full-text articles were further evaluated for eligibility. Eight studies were excluded because two of them reported acromioplasty only, four referred to the same study, and two did not provide quantifiable outcomes. Finally, six RCTs and one cohort study with a total of 651 patients met our inclusion criteria and were included in the meta-analysis.[22–28] The flow diagram of study selection is shown in Fig 1.

### Study characteristics and methodological quality

**Fig 2:**
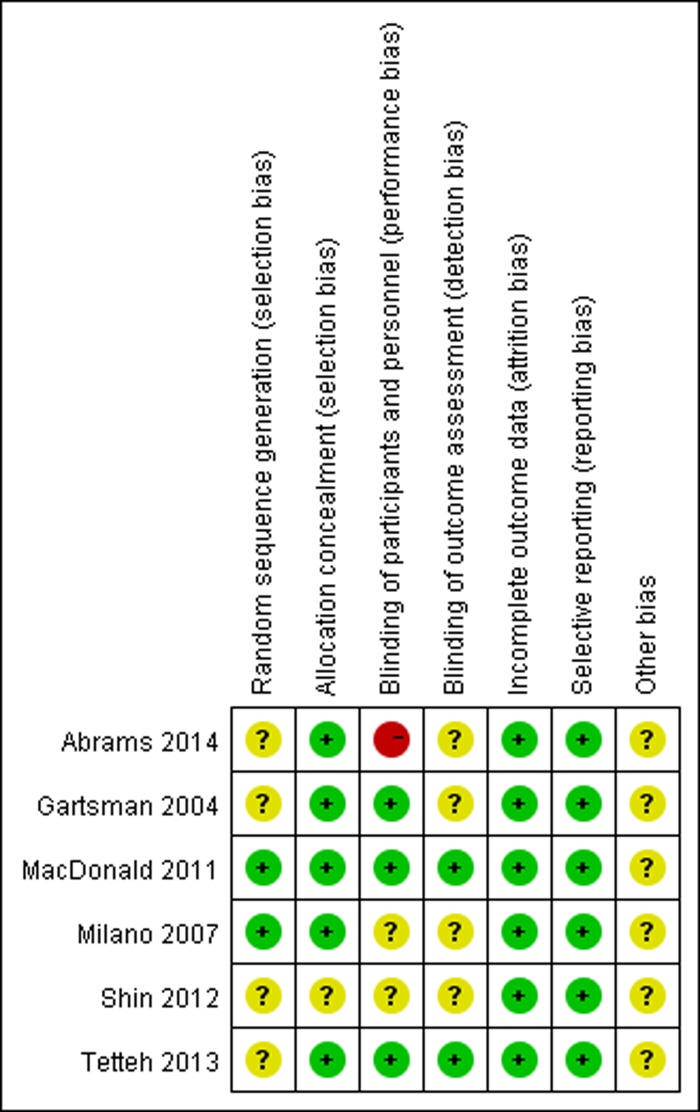
Summary of risk bias assessment. “+” = low risk of bias; “?” = unclear risk of bias; and “-” = high risk of bias.

Among the included RCTs, four studies were level I [22–24, 26, 27] and two were level II [25]. There was one study at high risk of bias in the blinding of participants and personnel, and the other studies were all at low risk or unclear (Fig 2). In addition, the NOS score of the cohort study was 8 [28], which was considered as a high-quality study. The experimental intervention were arthroscopic rotator cuff repair with acromioplasty in two studies, and arthroscopic rotator cuff repair with subacromial decompression in five studies. All trials were written in English. The characteristics of the included studies are presented in Table 1.

**Table 1:** The descriptive characteristics of included studies.

Study	Level of evidence	Study design	Inclusion criteria	surgical procedures	Sample size (male %)	Mean age in years	Mean follow-up in months	Follow-up Rate (%)	Study outcome measures
Gartsman et al. (2004)	I	RCT	Isolated, full-thickness supraspinatus tendon tear, and type II acromion	RCR versus RCR-SD	93 (55)	59.7	15.6 (12.3–18.9)	100	ASES
Milano et al. (2007)	I	RCT	Full-thickness rotator cuff tear, and type II, or III acromion	RCR versus RCR-SD	80 (55)	60.4	24	88.75	Constant score DASH Work-DASH
MacDonald et al. (2011)	I	RCT	Full-thickness rotator cuff tear ≤4 cm, and type I, II, or III acromion	RCR versus RCR-A	86 (65)	56.8	24	79.1	WORC ASES
Shin et al. (2012)	II	RCT	Full-thickness rotator cuff tear ≤3 cm and type I, II, or III acromion	RCR versus RCR-A	150 (56)	56.8	35	80	ASES Constant Score UCLA VAS
Tetteh et al. (2013)	I	RCT	Full-thicknessrotator cuff tear,and Type I, II, or III acromion	RCR versus RCR-A	61(64)	57.8	24	100	UCLA ASES SST
Abrams et al. (2014)	II	RCT	Full-thickness rotator cuff tear, and type I, II, or III	RCR versus RCR-A	114 (67)	58.8	24	83.3	ASES Constant Score UCLA VAS SST
Mardani-Kivi et al. (2016)	II	CS	Full-thickness rotator cuff tear, and Type I or II acromion	RCR versus RCR-A	67 (41.8)	56.7±8.7	27±4	100	SST Quick-DASH VAS

NOS Newcastlee Ottawa Scale, RCT randomized controlled trial, CS cohort study, ACR Arthroscopic cuff repair, RCR-A Rotator cuff repair with acromioplasty, RCR-SD Rotator cuff repair with subacromial decompression, ASES American Shoulder and Elbow Surgeons score, DASH Disabilities of the Arm, Shoulder, and Hand questionnaire, SST Simple Shoulder Test, UCLA University of California-Los Angeles score, VAS visual analog scale for pain, WORC Western Ontario Rotator Cuff Index, Work-DASH Work-Disabilities of the Arm, Shoulder, and Hand questionnaire, Quick-DASH Quick-disabilities arm shoulder and hand

### Clinical outcomes

**Fig 3:**
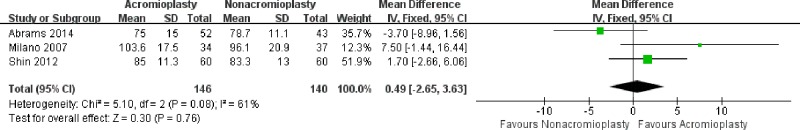
Forest plot of constant score between the treatment of acromioplasty and nonacromioplasty.

Constant score was reported in 3 RCTs [24,26,27], with 146 patients treated with acromioplasty and 140 with nonacromioplasty. The P value with the Cochran's Q test was 0.08, and the I^2^ statistic was 61%, which indicated high heterogeneity. Thus a random effect model was used. There was no statistically significant difference between the two group (MD, 0.49[95% CI, -2.65 to 3.63 ]; P = 0.76)(Fig 3).

**Fig 4:**
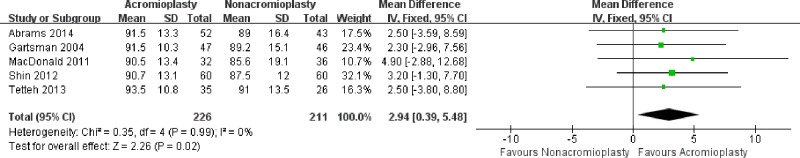
Forest plot of ASES score between the treatment of acromioplasty and nonacromioplasty.

ASES score was reported in 5 RCTs [22, 23, 25–27], with 226 patients treated with acromioplasty and 211 with nonacromioplasty. The P value with the Cochran's Q test was 0.99, and the I^2^ statistic was 0%, which indicated low heterogeneity. Thus a fixed effect model was used.There was statistically significant difference in favor of acromioplasty treatment(MD, 2.94[95%CI, 0.39 to 5.48]; P = 0.02)(Fig 4).

**Fig 5:**
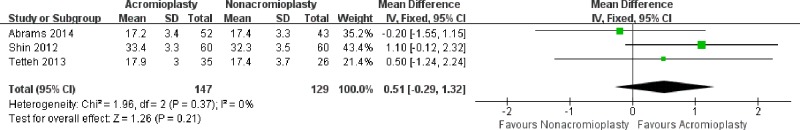
Forest plot of UCLA score between the treatment of acromioplasty and nonacromioplasty.

UCLA score was reported in 3 RCTs [25–27], with 147 patients treated with acromioplasty and 129 with nonacromioplasty. The P value with the Cochran's Q test was 0.37, and the I^2^ statistic was 0%, which indicated low heterogeneity. Thus a fixed effect model was used. There was no statistically significant difference between the two group (MD, 0.51[95%CI, -0.29 to 1.32]; P = 0.21)(Fig 5).

**Fig 6:**
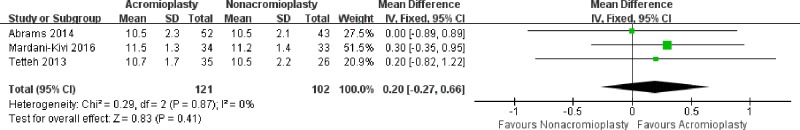
Forest plot of SST score between the treatment of acromioplasty and nonacromioplasty.

SST score was reported in 2 RCTs and one cohort study [25, 26, 28], with 121 patients treated with acromioplasty and 102 with nonacromioplasty. The P value with the Cochran's Q test was 0.87, and the I^2^ statistic was 0%, which indicated low heterogeneity. Thus a fixed effect model was used. There was no statistically significant difference between the two group (MD, 0.20[95%CI, -0.27 to 0.66]; P = 0.41)(Fig 6).

### Shoulder pain

**Fig 7:**
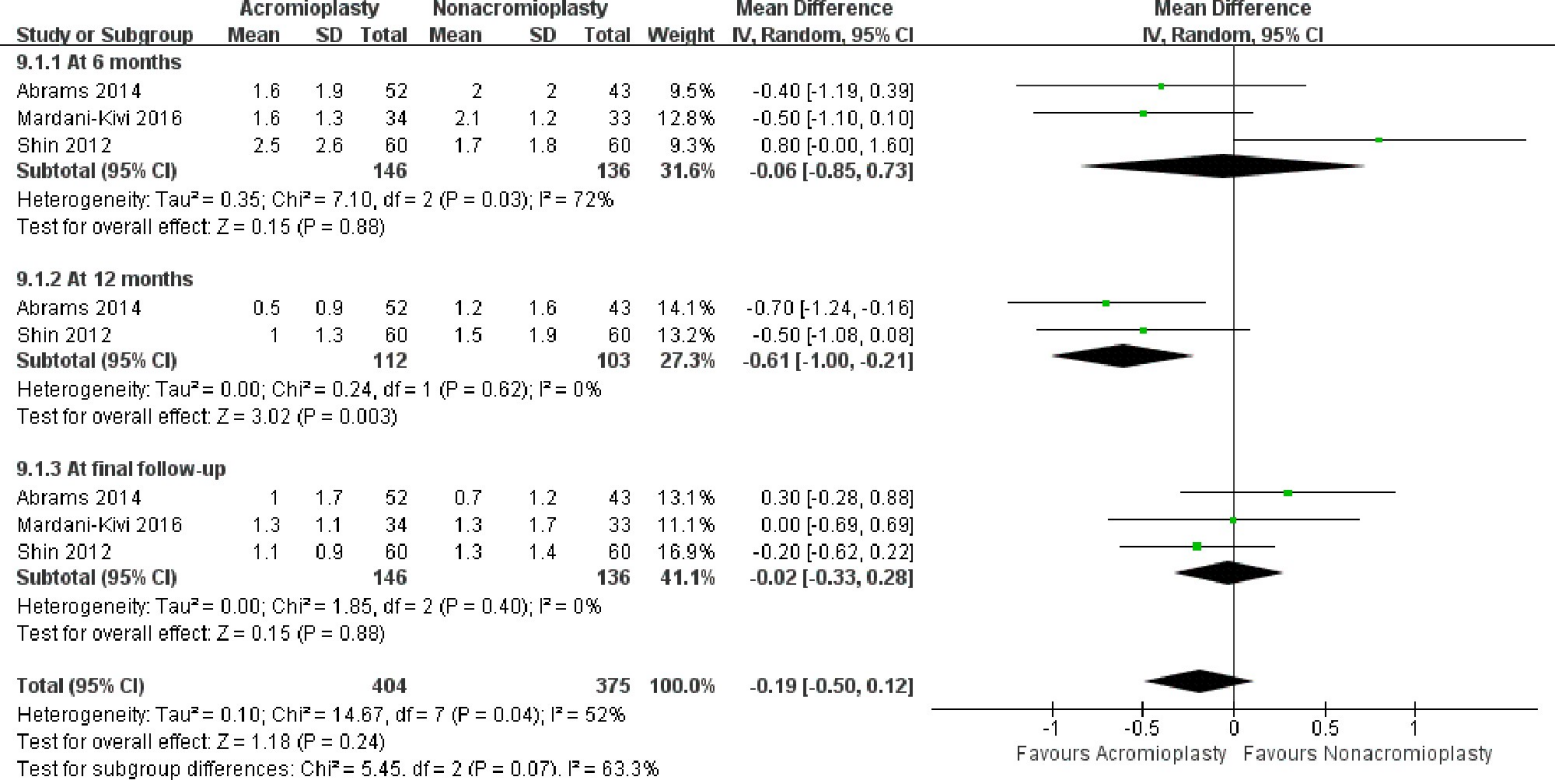
Forest plot of VAS score between the treatment of acromioplasty and nonacromioplasty.

At 6 months, 2 RCTs and one cohort study reported shoulder VAS score [26–28],with 146 patients treated with acromioplasty and 136 with nonacromioplasty, and there were no statistically significant difference between the two group (MD, -0.06[95%CI, -0.85 to 0.73], I^2^ = 72%, p = 0.88). At 12 months, the synthesis of 2 RCTs including 215 patients[26, 27] demonstrated a statistically significant difference in favor of acromioplasty treatment (MD, −0.61[95% CI, −1.00 to −0.21], I^2^ = 0%, p = 0.003). At the final follow-up, the pooled results of 2 RCTs and one cohort study [26–28] showed no statistically significant difference between the two group (MD, −0.02 [95% CI, −0.33 to 0.28], I^2^ = 0%, p = 0.88) (Fig 7).

### Reoperation rate

**Fig 8:**
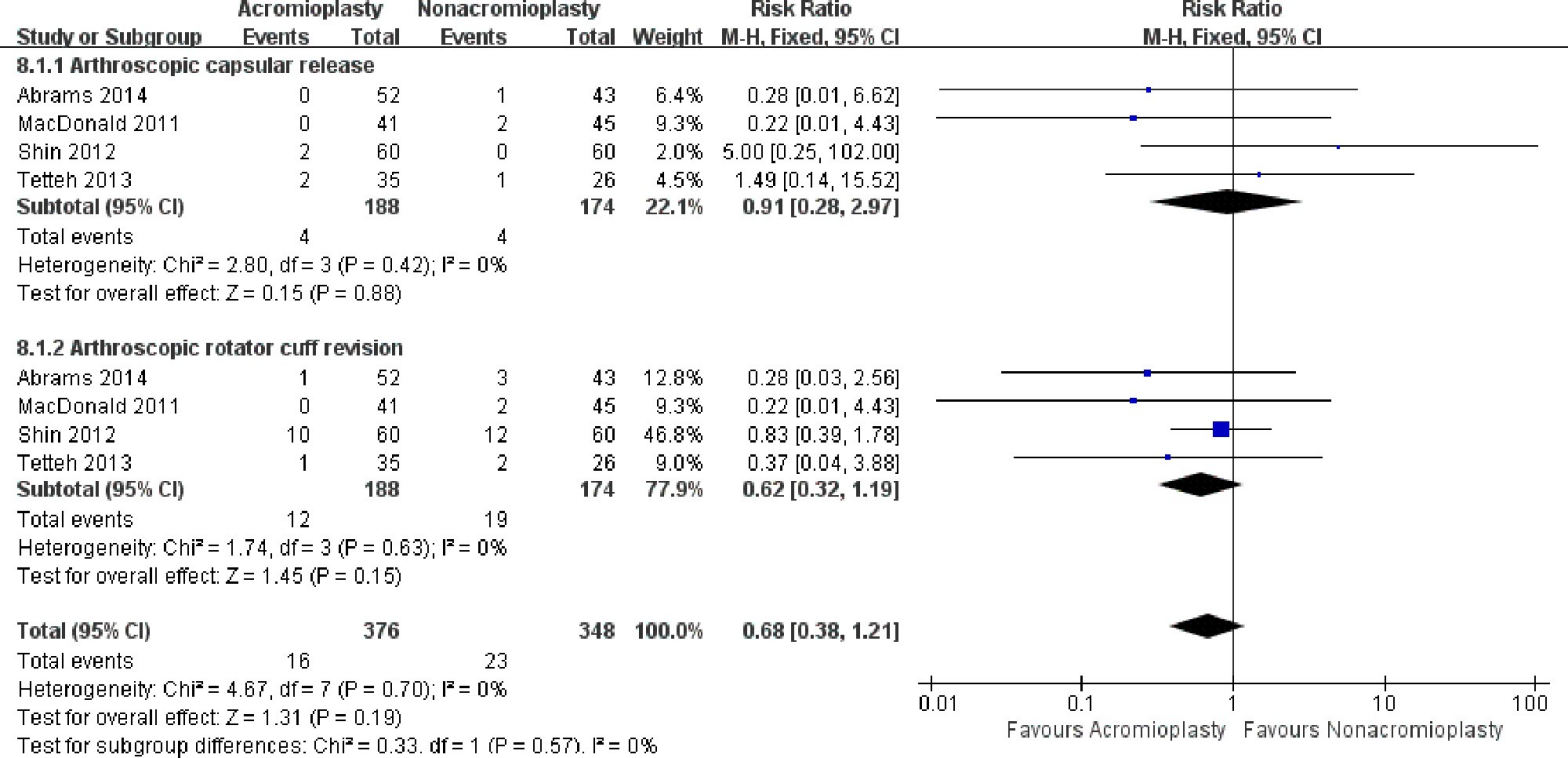
Forest plot of reoperation rate between the treatment of acromioplasty and nonacromioplasty.

Reoperation rate was reported in 4 RCTs [23, 25–27] including 188 patients treated with acromioplasty and 174 with nonacromioplasty. In the acromioplasty group, 4 patients in two studies [25, 27] had arthroscopic capsular release, and 12 individuals in three studies [25–27] had arthroscopic rotator cuff revision. There was no statistically significant difference between the two group (RR, 0.91[95%CI, 0.28 to 2.97]; I^2^ = 0%, P = 0.88). In the nonacromioplasty group, 4 patients in three studies [23, 25, 26] had arthroscopic capsular release, and 19 individuals in four studies [23, 25–27] had arthroscopic rotator cuff revision. The results also showed no statistically significant difference (RR, 0.62[95%CI, 0.32 to 1.19]; I^2^ = 0%, P = 0.15)(Fig 8).

### Sensitivity analysis

**Fig 9:**
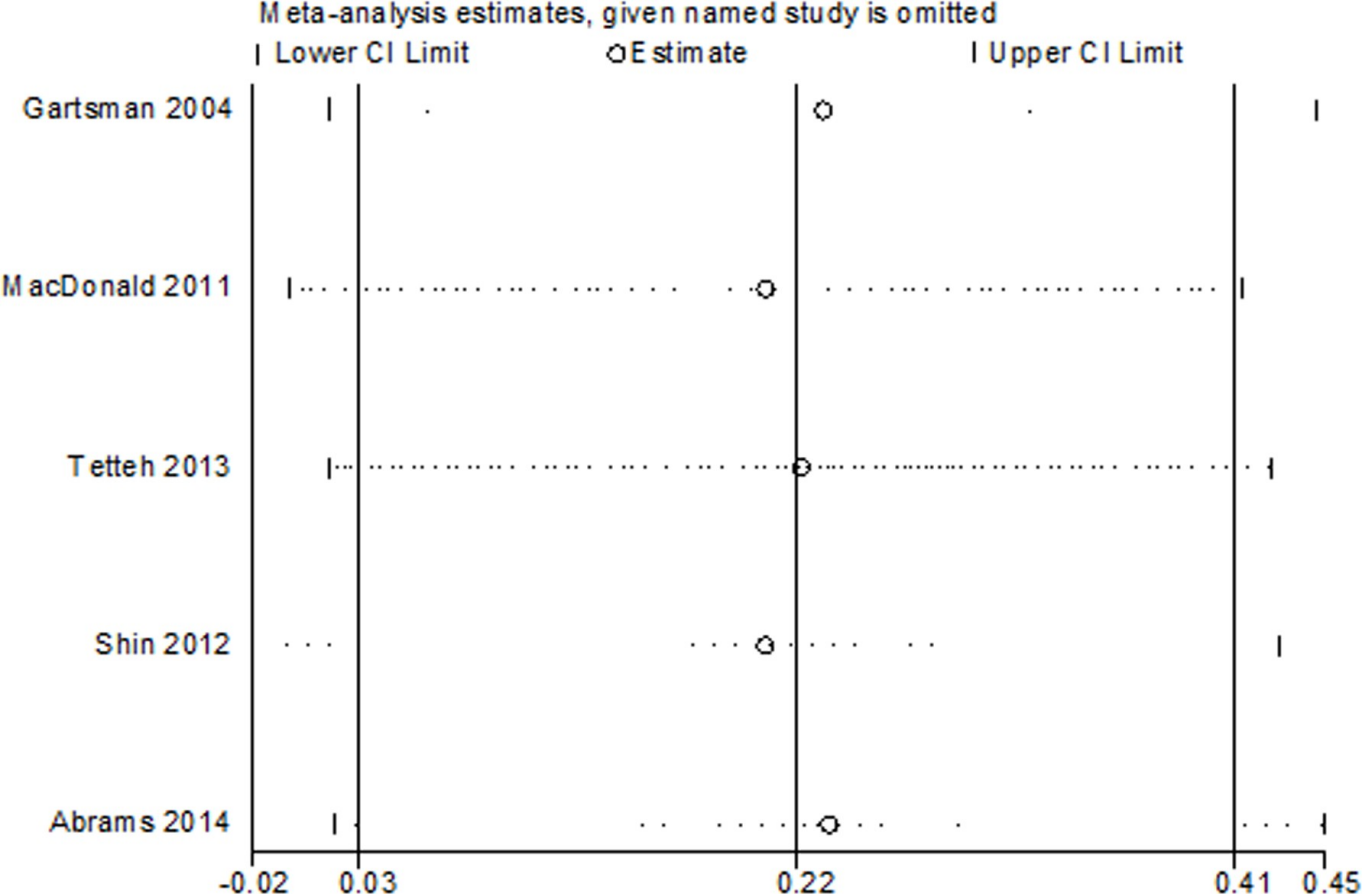
Sensitivity analysis of ASES score between the treatment of acromioplasty and nonacromioplasty.

Sensitivity analyses were performed by the leave-one-out approach from the aforementioned meta-analyses. There were no difference in the direction of the conclusions with studies removed in turn, which indicated that our results are statistically robust (Fig 9).

### Quality of the evidence and recommendation strengths

**Fig 10:**
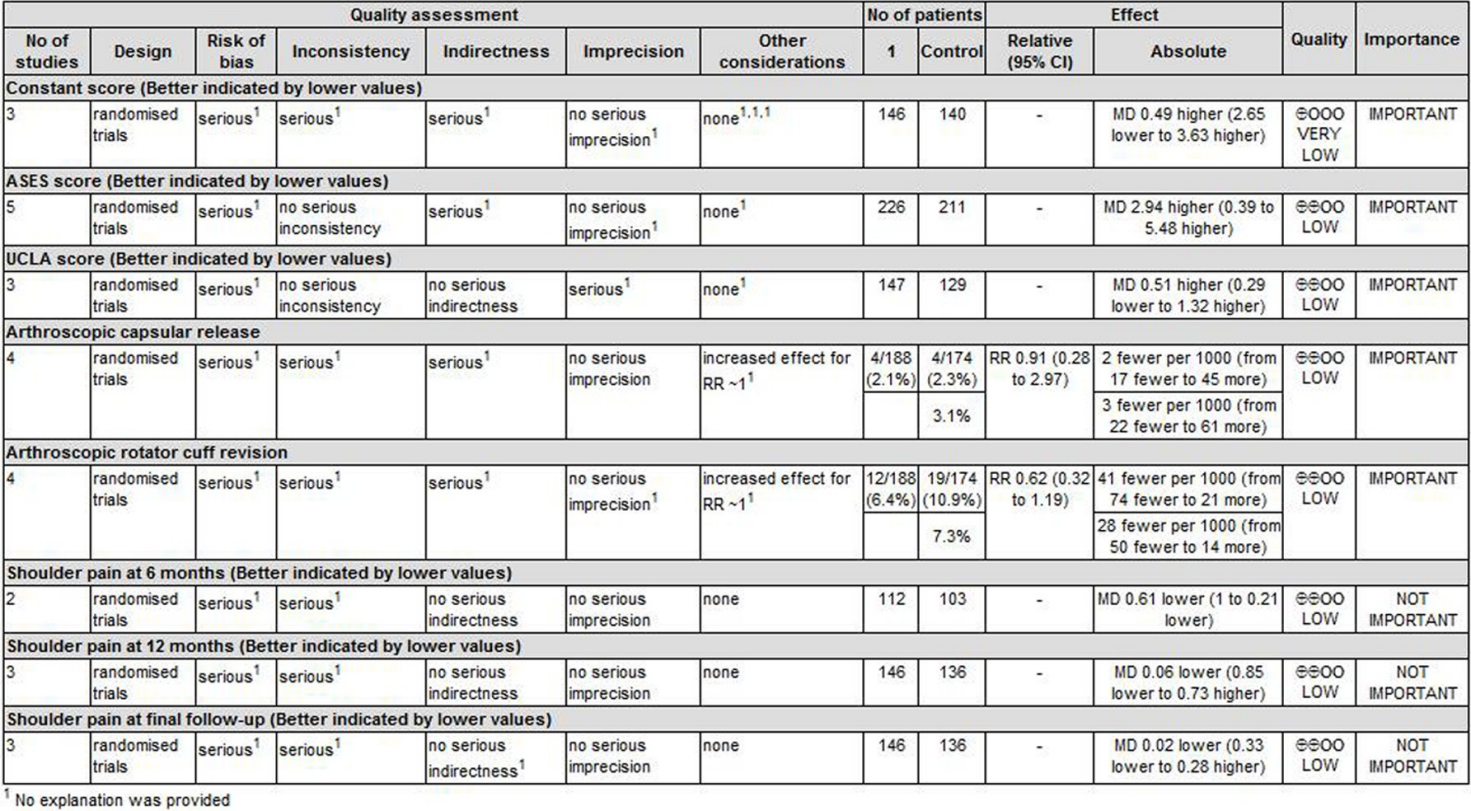
The GRADE evidence quality for main outcome.

Six outcomes in this meta-analysis were evaluated using the GRADE system. The evidence quality for each outcome was low (Fig 10). Therefore, we agree that the overall evidence quality is low, which indicates that further research is likely to significantly change confidence in the effect estimate and may change the estimate.

## Discussion

In our study, we first focused on subgroup analyses of shoulder pain and reoperation rate in patients undergoing arthroscopic rotator cuff repair with and without concomitant acromioplasty. We demonstrated that acromioplasty treatment was associated with a significant shoulder pain relief at 12 months postoperatively, but no significant difference in reoperation rate, which was mainly caused by rotator cuff retear and adhesive capsulitis. In addition, the routine use of acromioplasty significantly improved ASES score at the final follow-up. No significant differences were observed in other clinical outcomes (Constant score, UCLA score, SST score), or in pain relief at 6 months postoperatively and at the final follow-up. The overall evidence was low, which indicates that further research is likely to significantly change confidence in the effect estimate and may change the estimate. Based on the current available evidence, more high-quality studies are needed for further investigation.

Our study included one high-quality cohort study, 4 level I RCTs and 2 level II RCTs, which is a larger number of patients than previous systematic reviews and meta-analyses, making our results more dependable. Furthermore, the six RCTs had low risks of attrition bias and reporting bias, which contributed to reducing the systematic error. Another strength is that the heterogeneity, as assessed using the I^2^ statistic, was low across most outcome measures, indicating consistent outcomes across the studies. The outcome measure with relatively high heterogeneity was the Constant score. Notably, only three studies had a small number of patients in this analysis; thus, the results may require further confirmation.

We evaluated all of the available outcome measures, the number of which was larger than in previous reviews. Significant differences were observed in ASES and pain scores in the acromioplasty group. The mean difference in ASES score was 2.94 (from 0.39 to 5.48), and the mean difference in VAS score was -0.61 (from 1.00 to 0.21). However, a previous study of total shoulder arthroplasty showed that the minimal clinical relevant difference in ASES score was a 20.9-point improvement and for the VAS score a 1.4-point improvement [29]; thus, the significant differences of ASES score and pain score were not clinically relevant in our study. Moreover, only two studies with small numbers of patients were involved in the subgroup analysis of pain score, the result may be underpowered, and a definite conclusion could not be drawn on this topic.

Acromioplasty treatment is an essential part of ASD that consists of subacromial bursectomy and CAL resection. The importance of preserving CAL was first described by Codman et al. [30]. Then, several studies stated the potential disadvantages of the CAL resection, involving muscle weakness, adhesive capsulitis in the acromial space that limited shoulder mobility, and anterosuperior glenohumeral instability [31–33]. Rothenberg et al.[34] found the CAL played an important role in mechanosensory feedback loops that helped to dynamically stabilize the entire range of motion of the shoulder. Cay et al.[35] examined 40 patients undergoing rotator cuff repairs via magnetic resonance imaging and found that the acromio-humeral and coraco-humeral distances were narrower than normal limits in patients with rotator cuff tears. They concluded that the coracoacromial arch angle was an inducing factor for rotator cuff tears. Jaeger et al.[36] showed that patients with partial-thickness rotator cuff tears had satisfying clinical recovery to 90.9% of all cases after receiving ASD, and those with full-thickness tears had satisfying clinical recovery to 70.6%. Based on these findings, the efficacy of ASD and arthroscopic acromioplasty may be different in rotator cuff repair, and relevant studies comparing their functional effects would provide a better understanding of their differences. In addition, ASD and acromioplasty should be compared with nonacromioplasty [37]. Unfortunately, in our systematic review, ASD treatment was only reported in two of the included studies with different outcome measures; thus, there was no possibility of performing a pooled analysis of ASD. More well-designed studies should be subgrouped into ASD and acromioplasty.

The lesions of long head of the biceps (LHB), including tendinitis and partial tears, are a common source of pain and are generally associated with partial or complete rotator cuff tears, particularly in elderly patients. Tenotomy and tenodesis are widely used treatments of LHB[38]. Shin et al.[27] suggested that tenodesis with suture anchors be performed when the tendon tear involves more than 50% of the tendon thickness and the patient is less than 60 years of age; otherwise, a biceps tenotomy should be performed. However, whether LHB treatment is necessary in rotator cuff repair remains controversial. Shang et al.[39] and Watson et al.[40] concluded that concomitant tenotomy and tenodesis could relieve pain and improve functional outcomes of patients treated with repairable rotator cuff repairs. In contrast, Keong et al.[41] and Pander et al.[42] concluded that the LHB treatments did not positively speed recovery or affect outcomes. Upon comparing the LHB treatments, Godenèche et al.[43] reported that tenodesis renders better outcomes than tenotomy in isolated supraspinatus tears. Nevertheless, Shang et al.[39] found similar outcomes between the two treatments. In our meta-analysis, tenotomy and tenodesis were only reported in three studies, and no significant demographic differences were found between patients with and without acromioplasty. Moreover, the three studies demonstrated that acromioplasty did not significantly improve the clinical outcomes of rotator cuff repair. Thus, we could not assess the effect of concomitant LHB treatment in rotator cuff repair in our study.

Several limitations of our systematic review should be mentioned. First, numerous confounding factors, such as diversity of patient groups, clinical settings, surgical techniques and postoperative rehabilitations, may have affected the therapeutic results and led to potential biases. Second, the degree (tear size and number of tendons) of full-thickness rotator cuff tear was a critical factor affecting clinical outcomes. Two of the included studies[23,27] paid attention to the tear size of < 4 cm or < 3 cm, while the remaining five trials included a combination of all tear sizes. Only one study enrolled patients with an isolated, supraspinatus tendon tear, and the other trials reported two or more tendon tears. Under this circumstance, it is almost impossible to completely stratify the patients and perform generalizable analyses to determine the value of acromioplasty. Third, the outcome measures were not identical in each trial, potentially affecting the current findings of our study. Additionally, objective measures, such as preoperative and postoperative range of motion, strength testing, and radiographic assessment of rotator cuff, were not mentioned; thus, a comprehensive analysis could not be performed. Lastly, the clinical follow-up periods ranged from 12 to 35 months, and eligible studies with long-term follow-ups are required to consolidate the current findings.

## Conclusion

In summary, our present study demonstrated that the acromioplasty treatment is significantly superior to nonacromioplasty in shoulder pain relief at 12 months postoperatively and ASES score improvement at the final follow-up in conjunction with rotator cuff repair. However, these significant differences were not clinically relevant, and our meta-analysis included small sample size and limited number of eligible studies. Thus, there were no differences in shoulder function or pain scores for patients undergoing rotator cuff repair with and without acromioplasty. Further high-quality studies with larger sample sizes and long-term follow-ups are needed to clarify this issue.

## Supporting information

S1 Table PRISMA checklist of meta-analysis. (DOC)

S1 Appendix Search strategies. (DOCX)
