# Supplementary material for: Efficacy of concomitant acromioplasty in the treatment of rotator cuff tears: A systematic review and meta-analysis
Source: PLoS One. 2018 Nov 15;13(11):e0207306. doi: 10.1371/journal.pone.0207306 (PMC6237382; doi:10.1371/journal.pone.0207306)
Supplement: S1 Appendix — (DOCX) [file pone.0207306.s002.docx]

**S1 Appendix. Search strategy.**

**Cochrane Library:**

#1 MeSH descriptor: [Rotator cuff] this term only

#2 impingement or subacromial or acromion or degenearative or bony spurs:ti,ab,kw

#3 #1 or #2

#4 MeSH descriptor: [Shoulder] this term only

#5 shoulder:ti,ab,kw

#6 #4 or #5

#7 #3 or #6

#8 MeSH descriptor: [Acromioplasty] this term only

#9 depression or ligament resection or bursectomy or cuff repair:ti,ab,kw

#10 #8 or #9

#11 #7 or #10 Publication Year from 2000 to 2018
